# Outreach Through Facebook: Do Patients With Atopic Dermatitis Provide Clinically Relevant Information When Recruited for Surveys on Social Media?

**DOI:** 10.2196/45226

**Published:** 2023-10-05

**Authors:** Anne Sofie Frølunde, Susanne Thiesen Gren, Anne Grete Frøstrup, Peter Bo Poulsen, Anne Skov Vastrup, Christian Vestergaard

**Affiliations:** 1 Department of Dermatology Aarhus University Hospital Aarhus Denmark; 2 Department of Clinical Medicine Aarhus University Aarhus Denmark; 3 Pfizer Denmark Ballerup Denmark; 4 Atopisk Eksem Foreningen Copenhagen Denmark

**Keywords:** social media, atopic dermatitis, digital survey, recruit, patient perspectives, patient-reported outcomes, real-world data

## Introduction

Atopic dermatitis (AD) is a common chronic skin disease with a prevalence of up to 20% in children and up to 10% in adults [1]. There has been an increasing focus on the impact on the quality of life of the patients, family members, and caregivers [2], yet performing surveys to elucidate this is often laborious, time-consuming, and expensive. Social media has been used as a new platform for gaining insights into diseases through surveys, but for AD this has not been adequately tested. We have investigated the results obtained from a web-based survey recruiting respondents from a Facebook group hosted by the Danish Atopic Eczema Patients’ Organization (DAEPO) concerning whether it was representative of the population and if it returned relevant information on their disease.

## Methods

The survey consisted of 35 close-ended questions with checkboxes and was designed together with DAEPO. The inclusion criteria (self-reported) were being 18 years or older, previously or currently using a topical corticosteroid, and having been diagnosed with AD. No incentives were offered to participants. Data were collected anonymously between November 11 and December 22, 2021. Data collection and storage were compliant with European Union regulations on General Data Protection Regulation (GDPR). For the analysis, respondents were stratified into three groups according to severity as defined by the Patient Oriented Eczema Measure (POEM) instrument.

## Results

In total, 140 participants of 182 respondents met the inclusion criteria (recruitment rate 76.9%). Table 1 presents demographics, symptoms, and comorbidities. Table 2 shows health care use and disease management. Representativeness of survey responders (age, gender, and educational level) was investigated using chi-square test for goodness of fit, which confirmed the overrepresentation of younger female respondents with a higher educational level compared to the general Danish population. The limited participation of older adults has likewise been observed in previous studies using social media platforms [3]. Our data also indicated that the severity of AD correlated inversely with educational level in line with previous registry-based results, showing severe AD decreases the chance of completing higher education [4].

**Table 1:** Demographics and symptoms of respondents with atopic dermatitis (AD), stratified by disease severity.

				Mild (n=37)		Moderate (n=48)		Severe (n=55)		All (N=140)		*P* value (between AD severity group comparison)^a^	
**Age (years; N=140), n (%)^b^**													
	18-29			5 (14)		21 (44)		18 (33)		44 (32)		.01	
	30-39			9 (24)		8 (17)		17 (32)		34 (24)		.24	
	40-49			12 (32)		10 (21)		11 (20)		33 (24)		.33	
	≥50			11 (30)		9 (19)		9 (16)		29 (21)		.28	
Female (n=138), n (%)^b^				30 (83)		44 (92)		51 (94)		125 (91)		.20	
**Educational level (n=139), n (%)^b^**													.17
	Basic compulsory education^c^			0 (0)		1 (2)		7 (13)		8 (6)			
	Youth education^d^			13 (35)		20 (42)		24 (44)		57 (41)			
	Higher education^e,f^			23 (60)		25 (52)		22 (40)		69 (50)			
	Education not completed			1 (3)		0 (0)		0 (0)		1 (1)			
	Other			1 (3)		2 (4)		1 (2)		4 (3)			
**Age at diagnosis (years; n=139), n (%)**													
	0-2			14 (38)		19 (40)		31 (56)		64 (46)		.11	
	3-12			14 (38)		10 (21)		8 (15)		32 (23)		.04	
	**Older than 12 years^g^**												.33
		13-19	3 (8)		4 (9)		6 (11)		13 (9)				
		20-40	4 (11)		9 (19)		7 (13)		20 (14)				
		>40	2 (5)		5 (11)		2 (4)		9 (7)				
	Do not know			0 (0)		0 (0)		1 (2)		1 (1)		N/A^h^	
**Comorbidities (N=140), n (%)**													
	Asthma			18 (49)		16 (33)		24 (44)		58 (41)		.33	
	Allergic rhinitis			24 (65)		27 (56)		34 (62)		85 (61)		.71	
	Food allergies			12 (32)		15 (31)		24 (44)		51 (36)		.36	
	No asthma nor allergy			15 (41)		21 (44)		24 (44)		60 (43)		.67	
	Other types of allergies			5 (14)		9 (19)		7 (13)		21 (15)		.95	
**Bothersome symptoms (N=140), n (%)**													
	Itch			29 (78)		47 (98)		55 (100)		131 (94)		<.001	
	Dry skin			32 (87)		45 (94)		54 (98)		131 (94)		.08	
	Red rash			27 (73)		36 (75)		52 (95)		115 (82)		.008	
	Exudation			7 (19)		15 (31)		28 (51)		50 (36)		.005	
	Swelling			9 (24)		15 (31)		26 (47)		50 (36)		.06	
	Poor night’s sleep			6 (16)		15 (31)		39 (71)		60 (43)		<.001	
	Skin pain			16 (43)		22 (46)		41 (75)		79 (56)		.002	
	Other			0 (0)		6 (13)		5 (9)		11 (8)		.10	
	No symptoms			2 (5)		0 (0)		0 (0)		2 (1)		.06	

^a^Pearson chi-square test for AD severity group comparison. Significance level 5%.

^b^Chi-square goodness-of-fit test for responders’ representativeness with the Danish general population—age: χ^2^ goodness of fit=47.8, df=3, *P*<.001; gender: goodness of fit χ^2^_1_=87.9, *P*<.001; education: goodness of fit χ^2^_1_=13.8, *P*<.001).

^c^Basic compulsory education includes 9th and 10th grade.

^d^Youth education includes high school and vocational school.

^e^Higher education includes bachelor’s, master’s, or PhD programs.

^f^Pearson chi-square test for AD severity group comparison split between higher education or not.

^g^Pearson chi-square test performed for the AD severity group whose age at diagnosis was older than 12 years.

^h^N/A: not applicable.

**Table 2:** Management of disease, stratified by disease severity.

			Mild (n=37)		Moderate (n=48)		Severe (n=55)		All (N=140)		*P* value (between AD^a^ severity group comparison)^b^	
**Time since last AD consultation by dermatologist (n=139), n (%)**												.10
	Within last 6 months	13 (36)		16 (33)		32 (58)		61 (44)				
	Within last 6-12 months	4 (11)		7 (15)		3 (6)		14 (10)				
	Within last 1-2 years	9 (25)		9 (19)		6 (11)		24 (17)				
	More than 2 years ago	9 (25)		14 (29)		14 (26)		37 (27)				
	Never	0 (0)		2 (4)		0 (0)		2 (1)				
	Do not know	1 (3)		0 (0)		0 (0)		1 (1)				
**Use of moisturizers (n=137), n (%)**												.45
	Daily	31 (89)		40 (83)		50 (93)		121 (88)				
	At least once a week	2 (6)		3 (6)		3 (6)		8 (6)				
	Less than once a week	0 (0)		1 (2)		0 (0)		1 (1)				
	When needed	2 (6)		4 (8)		0 (0)		6 (4)				
	Never	0 (0)		0 (0)		1 (2)		1 (1)				
Moisturizers are recommended by the treating physician (n=137), n (%)			31 (89)		42 (89)		49 (89)		122 (89)		N/A^c^	
**TCS^d^ group, current or ever used (N=140), n (%)^e^**												
	Group I	17 (46)		22 (45)		22 (40)		61 (44)		.79		
	Group II	23 (62)		38 (79)		34 (62)		95 (68)		.12		
	Group III or IV	30 (81)		37 (77)		54 (98)		121 (86)		.004		
	Do not know which group	1 (3)		2 (4)		0 (0)		3 (2)		.33		
**Frequency of current TCS use (n=136), n (%)**												<.001
	Daily	3 (9)		8 (17)		22 (40)		33 (24)				
	3-5 times a week	5 (14)		16 (35)		18 (33)		39 (29)				
	1-2 times a week	9 (26)		11 (24)		6 (11)		26 (19)				
	1-3 times a month	5 (14)		6 (13)		3 (6)		14 (10)				
	Less than once a month	13 (37)		5 (11)		6 (11)		24 (18)				
**Periods of proactive use of TCS the last year (n=134), n (%)**												.05
	Less than 1 month	5 (15)		11 (24)		9 (17)		25 (19)				
	1-6 months	5 (15)		7 (15)		6 (11)		18 (14)				
	More than 6 months	5 (15)		12 (26)		24 (44)		41 (31)				
	No	19 (56)		16 (35)		15 (28)		50 (37)				
**Reactive use of TCS within the last month (n=131), n (%)**												<.001
	1 week	8 (25)		16 (36)		9 (16)		33 (25)				
	2 weeks	2 (6)		9 (21)		2 (4)		13 (10)				
	3 weeks	0 (0)		4 (9)		4 (7)		8 (6)				
	All last month	7 (22)		10 (23)		31 (56)		48 (37)				
	I have not used reactive treatment	15 (47)		5 (11)		9 (16)		29 (22)				

^a^AD: atopic dermatitis.

^b^Pearson chi-square test for atopic dermatitis severity group comparison. Significance level 5%.

^c^N/A: not applicable.

^d^TCS: topical corticosteroid.

^e^Pearson chi-square tests for AD severity group comparison for each of the TCS groups.

The majority of respondents had their AD diagnosis in childhood. Many across the three AD severity groups had asthma and allergy comorbidities. Symptoms such as itch, red rash, exudation, poor night’s sleep, and skin pain were significantly more frequent among patients with severe AD (Table 1). The frequency of dermatology visits did surprisingly not differ significantly between severity groups, whereas reactive treatment use patterns in the last month were observed significantly more in those with severe AD (Table 2). This indicates that many patients with AD are not adequately treated and should have closer contact with the health care system to receive timely and optimal treatment.

## Discussion

In conclusion, we found that social media may be used for disease surveys, although with a risk of lack of representativeness of the general population (ie, favoring those who are female, younger, and well educated). With this in mind, however, outreach through social media is an easy cost-effective way of acquiring a large amount of information and may be a useful platform to obtain relevant disease information on patient-reported outcomes for patients with AD, and female patients in particular.

## Abbreviations

AD: atopic dermatitis
DAEPO: Danish Atopic Eczema Patients’ Organization
GDPR: General Data Protection Regulation
POEM: Patient Oriented Eczema Measure
